# Perceptions of followership among nurses: A qualitative study

**DOI:** 10.1016/j.ijnsa.2024.100222

**Published:** 2024-07-17

**Authors:** Sulaiman Alanazi, Richard Wiechula, David Foley

**Affiliations:** a College of Nursing, Jouf University, Jouf, Saudi Arabia; b Adelaide Nursing School, Faculty of Health and Medical Sciences, University of Adelaide, Adelaide, Australia

**Keywords:** Leadership, Nurses, Qualitative research, Saudi Arabia

## Abstract

**Background:**

Followership is defined as the role individuals play in supporting, contributing to, and realizing the vision and directives set by their leaders. Such a role is indispensable in healthcare, facilitating effective team dynamics and healthcare delivery. Within the nursing field, it encompasses nurses' active engagement and participation in healthcare delivery, ensuring safety, fostering teamwork, and enhancing patient outcomes. Despite its significance, the exploration of followership within the nursing context of Saudi Arabia remains limited.

**Objective:**

This study aims to explore how followership is perceived and practiced by nurses in this unique cultural and professional setting, and its implications for healthcare delivery.

**Methods:**

We conducted a qualitative inquiry involving seven registered nurses working in hospitals affiliated with the Saudi Arabian Ministry of Health. Semi-structured interviews were conducted, and a thematic analysis was utilized to extract key findings.

**Results:**

Our thematic analysis identified four main themes and several sub-themes that encapsulate the participants' perspectives on followership. The themes include: (1) Understanding of followership, where a predominant lack of clarity about the concept was observed, often conflating it with teamwork; (2) Followers' involvement in decision-making, highlighting the limited participation of nurses in decision-making processes due to hierarchical and autocratic leadership structures; (3) Barriers to followership, which encompassed issues such as poor leadership, the undervaluation of the follower role, lack of training and development opportunities, challenges in collaboration, and language barriers; and (4) Facilitators of followership, identified as effective leadership, followership training, communication skills, positive relationships, respect, collaboration, understanding of roles, commitment, and flexibility. These findings elucidate the complex landscape of followership within the nursing profession in Saudi Arabia, revealing both the challenges and pathways to fostering effective followership in healthcare settings.

**Conclusion and Implications:**

This study reveals a widespread lack of awareness about followership among nurses in Saudi Arabia, highlighting significant challenges related to hierarchy and the undervaluation of the follower role in nursing practice and education. It underscores the need for educational and training interventions that redefine and elevate the role of followership in clinical settings to enhance collaboration, assertiveness, and decision-making skills. Moreover, the study advocates for the re-evaluation of leadership practices to better acknowledge and value followership, promoting a more flattened hierarchy that encourages active participation in patient care and organizational development. Implementing these changes could improve patient outcomes and increase nurse satisfaction by effectively addressing the identified barriers related to hierarchy and leadership.

**What is already known about this topic?**

• Effective health care teams require exemplary followers.
• Followership is an important role for nurses.
• Followership hasn't received sufficient theoretical and empirical attention.

Alt-text: Unlabelled box

**What this paper adds:**

• Followership is not understood and is often merged with teamwork and other work-related concepts.
• The study found that in cultures characterized by high power distance, nurses tend to undervalue the role of a follower.
• The practice of exemplary followership is reliant on the type of leadership in the workplace.

Alt-text: Unlabelled box

## 1 Background

Followership, as conceptualized in organizational behavior, refers to the behaviors, processes, and roles exhibited by individuals who are considered followers within the context of leadership dynamics (Kellerman, 2008; Crossman and Crossman, 2011). It encompasses the active engagement, contribution, and influence of followers in relation to their leaders and the overall organizational environment (Kelley, 1988; Chaleff, 2009). A distinctive aspect of the concept of followership is assertiveness and the ability of followers to constructively challenge leadership when necessary to ensure adherence to organizational objectives and ethical standards (Kelley, 1988; Chaleff, 2009). Despite its significance, followership has historically been under-researched compared to leadership (Carsten et al., 2010; Spriggs, 2016; Loyola and Aiswarya, 2023) an imbalance that can be attributed to two main factors. First, the conventional perspective on leadership has predominantly highlighted the leader's role as the primary determinant of an organization's success or failure, thereby marginalizing the critical importance of followers in the leadership equation (Martin, 2015; Honan, Lasiuk, and Rohatinsky, 2022; Loyola and Aiswarya, 2023). This leader-centric view has contributed to a lack of attention towards the role and impact of followership in organizational dynamics. Second, there exists a stereotypical assumption that followers are passive participants within the leadership process, thereby diminishing the perceived value of their contributions and experiences (Riggio, 2020; Honan, Lasiuk, and Rohatinsky, 2022; Loyola and Aiswarya, 2023). This has led to a significant oversight in understanding the active and dynamic roles followers play in influencing leadership outcomes and organizational success (Loyola and Aiswarya, 2023).

However, Kelley, through his seminal work “In praise of followers” (1988), pioneered a shift in perspective on followership, challenging traditional views by introducing a model that emphasized the active and critical role of followers in organizations. Kelley's followership model has had a profound impact on leadership studies, shifting the focus from a leader-centric perspective to a more balanced view that recognizes the dynamic interplay between leaders and followers. It highlights the importance of followers who are not just passive recipients of leadership but active participants in shaping organizational outcomes (Alanazi, Wiechula, and Foley, 2023). This model has paved the way for further research into followership, encouraging scholars and practitioners alike to explore the ways in which followers can be empowered and engaged to contribute more effectively to their organizations. As a result, the field of followership research is gaining momentum, with a growing body of work focused on understanding the roles, characteristics, and experiences of followers across various organizational sectors, including healthcare (Alanazi, Wiechula, and Foley, 2023).

In healthcare, the critical nature of patient care and safety amplifies the importance of followership (Andersen et al., 2010). Effective followership involves assertiveness, active engagement, critical thinking, and a commitment to both support and constructively challenge leadership, alongside adhering to ethical standards (Kelley, 1992; Chaleff, 2009). These traits are crucial for fostering a culture of safety and enhancing communication within healthcare teams (Hinshaw, 2016; Hay-David et al., 2022). Furthermore, such followership leads to improved team dynamics, reduced errors, and better patient outcomes by creating an environment where team members feel empowered to share insights, voice concerns, and participate in decision-making (Whitlock, 2013; Adams and Gibson, 2024). The active participation and independent critical thinking inherent in positive followership styles also result in increased job satisfaction (Leung et al., 2018), reduced burnout (Crawford and Daniels, 2014), and enhanced workplace performance in healthcare settings (Sculli et al., 2015). In contrast, ineffective followership, which is characterized by a reluctance to question others' actions, when necessary, can jeopardize patient safety and increase the likelihood of errors in clinical settings (Fadden and Mercer, 2019; Whitlock, 2013; Bould et al., 2015; Green et al., 2017). The Elaine Bromiley case exemplified the potential negative impact of ineffective followership on patient outcomes (Bould et al., 2015; Green et al., 2017). Elaine Bromiley incurred hypoxic brain injury as a result of a delayed surgical airway prior to elective surgery. The perioperative nursing staff were aware of the necessity to perform a surgical airway procedure to preserve the patient's life. Nonetheless, they were reluctant to communicate their concerns to the anesthetists who were distracted while attempting to perform an oral intubation (Bould et al., 2015; Green et al., 2017). In fact, many clinical practice errors can be attributed to human factors, such as ineffective communication, leadership, or followership (Hinshaw, 2016; Fadden and Mercer, 2019; Whitlock, 2013; Bould et al., 2015; Green et al., 2017). Therefore, developing a comprehensive understanding of followership in healthcare is crucial, especially among nurses, who represent the largest group within healthcare systems (Freeman, 2020; Abdel-Malak, 2016; Lopez and Freeman, 2018).

Despite the growing body of research on followership, it has been predominantly centered on Western contexts (Chaleff, 2016; Kelley, 2008; Can and Aktaş, 2012; Uhl-Bien et al., 2014). This gap highlights the need for studies that explore followership in varied cultural and organizational contexts, such as Saudi Arabia, a cultural context characterized by high power distance and hierarchical organizational structures (Hofstede, Hofstede, and Minkov, 2010; Almalki, FitzGerald, and Clark, 2011). In cultures of this nature, hierarchy is usually emphasized, independent thinking is discouraged, and followers are more dependent on leaders (Schuder, 2016). By examining followership within such contexts, research can offer valuable insights into how different cultural and organizational environments influence perceptions, behaviors of followership, and, consequently, the outcomes of teams and organizations (Chaleff, 2016; Kelley, 2008; Carsten et al., 2010).

This qualitative study, the latest component of a larger mixed methods research project, specifically aims to delve into the concept of followership within the nursing context of Saudi Arabia. Building upon the foundational work of our scoping review (Alanazi, Wiechula, and Foley, 2023), which identified and mapped the existing literature on followership among healthcare clinicians, and a subsequent quantitative analysis (Alanazi, Wiechula, and Foley, 2022) that explored the diverse followership styles of nurses in Saudi Arabia with relation to their sociodemographic profiles, this phase seeks to illuminate the nuanced perceptions and enactments of followership in this unique cultural and professional setting. The culmination of these efforts aims to contribute to the development of effective leadership and followership training programs, tailored to meet the needs of diverse healthcare environments worldwide, thereby enhancing team dynamics and improving patient care on a global scale.

## 2 Methods

### 2.1 Study design

This study employed a qualitative methodology, with semi-structured interviews, to comprehend how nurses conceptualize their roles within organizational contexts. Thematic analysis was used to explore participants’ comprehension of followership, perspectives on the attributes of effective followers, and the factors that promote or impede effective followership within their organizations. This technique is appropriate for conducting exploratory research because it has the potential to increase the depth and reveal the thinking of participants that quantitative research methods might not have been able to capture (Bryman, 2004; Conger, 1998).

### 2.2 Participants

The study was carried out in Saudi Arabian public hospitals that are affiliated with the Ministry of Health. In Saudi Arabia, the Ministry of Health is responsible for providing nearly 80 % of all healthcare services (Ministry of Health in Saudi Arabia, 2020). The recruitment process for the study targeted nurses working in these settings using convenience sampling. Semi-structured interviews were conducted with seven registered nurses from four regions in Saudi Arabia working in five hospital departments including surgery, mental health, emergency, intensive care unit, and a post-anesthesia care unit. Five participants were staff nurses and two participants were head nurses. Five of the participants were male, with two female nurses. Six of the nurses were Saudis, with one nurse was an expatriate. The mean duration of employment for respondents with their present organizations was 5.8 years.

In our study, data saturation was thoughtfully considered and achieved with seven nurses from diverse healthcare settings in Saudi Arabia. This was determined through a meticulous analysis process where no new themes emerged from the data by the final interviews, indicating that the collected data were adequate for conducting the thematic analysis. The guiding questions for these interviews were informed by the findings from the quantitative component of our study (Alanazi, Wiechula, and Foley, 2022), and designed to explore various dimensions of followership, including personal definitions, clinical shift routines, interactions with other team members, perceived roles and responsibilities, participation in decision-making, views on and preferences for leader or follower roles, challenges encountered, experiences, and the attributes of effective followership from the participants' perspectives. This approach not only enriched our data collection but also ensured thematic saturation, offering a depth and breadth of understanding of the topic, and allowing for a substantial volume of findings from a relatively small number of participants.

### 2.3 Procedure

The principal investigator sent an email to the General Administration of Nursing at the Ministry of Health detailing the study and requesting they invite nursing staff from all Ministry of Health's hospitals. Seven participants expressed interest. Interviews, ranging from 25 minutes to an hour, were scheduled at the participants' convenience, conducted via Zoom࣪, and video recorded with informed consent. These semi-structured interviews explored many aspects relevant to followership as described in the above section. Though healthcare communication in Saudi Arabia primarily uses English (Almalki, FitzGerald, and Clark, 2011), the protocol was available in both Arabic and English. Three interviews were in Arabic and later translated to English; the rest were in English. All interviews were transcribed verbatim for coding.

### 2.4 Coding and data analysis

The interviews underwent qualitative coding using the six-step thematic analysis approach developed by Braun and Clark (2006). Qualitative methods scholars suggest that thematic analysis is advantageous due to its flexibility, which allows it to be tailored to suit the requirements of qualitative studies (King, 2004; Nowell et al., 2017). It is also capable of generating a comprehensive and detailed representation of data (Braun and Clark, 2006; King, 2004; Nowell et al., 2017). The initial stage involved closely examining the transcripts through repeated readings to become familiar with the data (Braun and Clark, 2006). In the second stage, initial codes were generated by coding relevant data segments related to the research topic of followership (Braun and Clark, 2006). The third stage involved identifying themes by scrutinizing and categorizing the codes into broader themes relevant to followership (Braun and Clark, 2006). In the fourth step, the preliminary themes were refined and developed while gathering and consolidating all data related to each theme (Braun and Clark, 2006). Then in the fifth step, a thorough examination assessed the relevance and connections between subthemes and main themes, as well as the interrelationships among the themes (Braun and Clark, 2006). Finally, in the last stage, the findings were synthesized and reported (Braun and Clark, 2006).

### 2.5 Trustworthiness

To ensure rigor in this qualitative research, we considered the principles of trustworthiness outlined by Lincoln and Guba (1985), which include credibility, transferability, dependability, and confirmability. The participants' responses were recorded in full, transcribed verbatim, and Arabic interviews were carefully translated to ensure the credibility of the subsequent analysis. This process provided comprehensive and detailed descriptions of the research context, settings, procedures, and results, thereby satisfying the principle of transferability. To adhere to the principles of dependability and conformability, the principal investigator's supervisors actively participated throughout the analysis process to ensure that the findings were grounded in the participants' perceptions and that the participants' perspectives were accurately reflected in the final report.

### 2.6 Ethical considerations

The study received approval from the University of Adelaide Human Research Ethics Committee (H‐2020‐026) and the Central Institutional Review Board at the Ministry of Health in Saudi Arabia (20‐161E). Before interviews, participants were briefed about the study and provided informed consent, allowing recordings. All data was anonymized. Participation was voluntary, with the option to withdraw at any stage. Voice recordings and transcripts were securely encrypted and stored on a password-protected computer.

## 3 Results

**Fig. 1.**
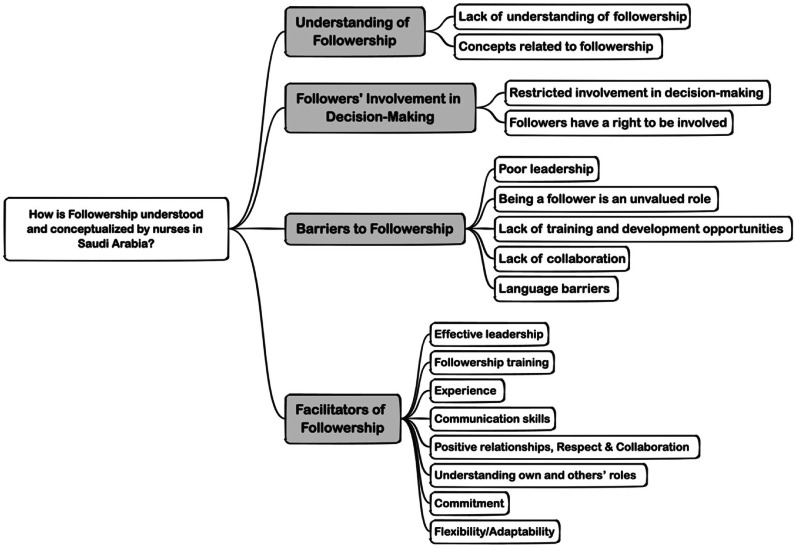
Themes and sub-theme.

The study's results revealed the identification of four major themes, accompanied by several sub-themes, through which nurses in Saudi Arabia have approached the concept of followership. These themes are “understanding of followership,” “followers’ involvement in decision-making,” “barriers to followership,” and “facilitators of followership,” see Fig. 1.

## 4 Understanding of followership

The first theme addressed participants' understanding of followership. Most were not familiar with the term followership and had difficulty finding a specific definition. Some did, however, articulate some concepts they felt could be related to followership. Two sub-themes were developed: Lack of understanding of followership and concepts related to followership.

### 4.1 Lack of understanding of followership

The term 'followership' was largely unfamiliar to the participants, underscoring a significant challenge in grasping its fundamental principles. Most participants associated followership predominantly with passive obedience rather than active engagement in leadership dynamics. This trend suggests a superficial understanding, where followership is perceived merely as following orders, potentially indicating a lack of deeper, critical engagement with the concept.

*To be honest, no. I didn't get the meaning of it. But followers are like the people who follow the leader (Participant 4).*

*I have not heard of it before, but in my opinion, it relates to the work team and their strategies to coordinate work, following leaders and guidelines (Participant 6)*

Further discussions revealed a consistent pattern among participants to equate followership with basic teamwork or other operational aspects of their roles. This conceptual limitation became more pronounced when participants discussed barriers and facilitators of effective followership. Rather than identifying factors intrinsically linked to followership qualities, participants frequently associated it with general workplace challenges such as work-life balance, long shift hours, understaffing, and time management.

*sometimes, problems outside the scope of work significantly affect the nurse and his performance at work (Participant 5).*

*long working hours sometimes affect focus…and this is one of the important obstacles that affect the effectiveness of subordinates (Participant 7).*

These responses collectively illustrate a narrow interpretation of followership, primarily focusing on obedience and task execution.

### 4.2 Concepts related to followership

Despite their unfamiliarity with the term 'followership,' in some instances, participants demonstrated an intuitive understanding of its critical aspects, which include decision-making, support for leadership, and the nurturing of workplace relationships. These insights collectively suggest that participants, albeit unconsciously, recognize the importance of followership in enhancing organizational dynamics and effectiveness.

One participant associated followership with effective team decision-making, noting its crucial role in collective problem-solving and operational efficiency within teams. This reflects an understanding that followership involves active engagement in the decision-making process, enhancing team functionality.

*But as a concept, it's how to be effective and how to make decisions with the team to be able to do your work in a professional way (Participant 1).*

Another participant highlighted the relational aspect of followership, emphasizing its significance in supporting leadership. This view portrays followership as a supportive partnership that is vital for those in non-leadership roles, enhancing the leadership process by reinforcing and executing decisions.

*But I think followership is the partner of leadership and focuses more on the role of the staff, not the leader (Participant 2).*

Additionally, the influence of followership on interpersonal relationships within the workplace was noted, indicating its broader implications beyond hierarchical interactions. This perspective shows an awareness of the social dynamics of followership, recognizing its impact on the quality of interactions among all organizational members.

*I believe that it relates to the relationship between employees and co-workers and the relationship between the boss and subordinates (Participant 7).*

## 5 Followers’ involvement in decision-making

The study participants were asked about their participation in decision-making regarding patient care and the extent of their involvement in this process. They described that their engagement in the decision-making process was primarily through the articulation of their opinions on the provision of healthcare services to patients. However, they expressed that, as followers, their participation in decision-making is limited by hierarchy and power disparities, but they did recognize the right to be fully involved, as explained in the following sub-themes.

### 5.1 Restricted involvement in decision-making

Hierarchical structures within organizations significantly limit the participation of nurses in decision-making, underscoring a rigid communication barrier with higher levels. This restriction often forces nurses to operate strictly within the confines established by their immediate supervisors, avoiding direct communication with upper management unless absolutely necessary. This dynamic illustrates the challenges nurses face in navigating organizational hierarchies, where stepping outside established lines of communication can lead to significant consequences.

*We can't communicate with the higher levels of the hospital. If, for example, I contact the nursing department without permission from my head nurse, a big problem will happen. So, unless the situation is complicated, I will not pass my head nurse to communicate with the higher level (Participant 4).*

Experiences of direct authority imposition also highlight the top-down approach in decision-making processes within the participants’ health organizations, where the orders from superiors are not to be questioned. This reflects a decision-making culture where orders are expected to be executed without input, emphasizing the autocratic nature of leadership in some settings.

*His reaction was from the premise that he is the boss and you must accept this matter, you must admit the patient even if he was carrying an infection, I mean, I am your boss, do what I tell you (Participant 5).*

Furthermore, the dominance of physicians over nurses in making patient care decisions was pointed out, illustrating the traditional roles within medical hierarchies where doctors often have the final say. This participant's comment reveals the perceived marginalization of nursing input in clinical decisions, reinforcing the structured power dynamics within healthcare.

*The decision-making in relation to patient care is not all up to nurses or nursing in general. So that's more up to the doctors, to be honest with you (Participant 2).*

*Do you mean the leader? In this case, the doctor (Participant 6).*

These insights collectively depict an organizational culture that marginalizes nurses in decision-making processes, especially in patient care, which poses systemic challenges due to rigid hierarchical structures and entrenched traditional roles.

### 5.2 Followers have a right to be involved

Participants highlighted their critical role in decision-making, rooted in their proximity to patients and firsthand knowledge of patient needs. They stressed that their unique insights are indispensable for informed healthcare decisions.

*I think that the role of the nurse is very important in making decisions because the nurse is the person who is very close to the patient… You must express your opinion… in the end, this is your patient whom you are obligated to during the shift, it is your responsibility (Participant 5).*

Illustrating the significance of nursing input, one nurse described how a proactive suggestion during a resuscitation led to a life-saving outcome.

*I remember that I was in the resuscitation team for a woman, and after the end of the 30 minutes, the doctor announced stopping the resuscitation process, but I suggested that we continue for an additional 10 or 15 minutes because we would not lose anything. The doctor agreed to that, and after 10 minutes had passed, the pulse returned and the patient was intubated and transferred to the intensive care unit, and everyone was happy (Participant 6).*

Nurses also advocated for their right to challenge management decisions based on their clinical experience, especially when they believe a different approach may benefit the patient.

*Even if the doctor orders this medication or this management, and my previous experience has shown that the management will not benefit the patient, so I will give, for example, my feedback or my previous experiences with regards to the certain management that the doctor wants to do for the patient (Participant 3).*

Despite these insights, nurses often face hierarchical resistance that diminishes their involvement in decision-making.

## 6 Barriers to followership

This theme focuses on barriers to effective followership, incorporating only those barriers identified by participants that directly impact nurses' ability to function as effective followers. While participants mentioned various factors, we have selectively included those most pertinent to followership based on our analysis. Notably, some barriers - such as work-life balance, long working hours, and understaffing - were discussed under the initial theme 'Understanding of followership.' These were not emphasized in this section due to their less direct relevance to followership, a distinction stemming from participants' limited understanding of the concept. Their mention in the 'Understanding of followership' theme served to illustrate this limited understanding by showing how participants associated these broader workplace factors with followership.

### 6.1 Poor leadership

Analysis of participant feedback revealed a profound concern about the effects of leadership quality on nursing performance and patient care. A critical aspect highlighted involves the allocation of responsibilities. Participants noted that leaders often lack a deep understanding of their team's roles, skills and limitations, which can lead to mismanagement and errors.

*The doctor is also involved in the occurrence of this error. It is assumed that the doctor should not give an order to a student. The doctor did not expect that the student would give a completely different drug from what the doctor described (Participant 6).*

Moreover, the disregard for nurses' inputs by some leaders exacerbates the challenges within healthcare settings. This often results in missed opportunities to improve patient outcomes and may lead to serious healthcare failures.

*Instead of curing something simple, a major deterioration in the patient's condition was the result of the doctor or manager not listening to his subordinates (Participant 6).*

The personal and professional repercussions of poor leadership are profound. Participants expressed frustration and disappointment when their contributions were overlooked or undervalued, leading to a loss of trust and respect for their leaders.

*you will have a negative impression of this leader, since you came to him with an opinion or an idea, and he refused or did not take the issue into due consideration. In this case, you do not come to him again, my dignity prevents me from presenting him with an idea or suggestion again (Participant 5).*

### 6.2 Being a follower is an unvalued role

The inclination towards leadership roles among participants underscores a pervasive undervaluation of followership within professional environments. This trend is indicative of a broader organizational culture that elevates the status of leaders while marginalizing the roles of followers.

Discussions about professional roles in healthcare predominantly highlight leadership, sidelining followership and its significance. This neglect is encapsulated by sentiments that followership is rarely discussed or valued.

*No one is talking about followership; actually, we hear only about leadership (Participant 2).*

Such an environment leads to the exclusion of followers from critical developmental and support opportunities, despite their integral role in healthcare systems.

*Most of what we talk about is the role of the boss, the subordinates feel that they are absent from the training courses, absent from many things despite their importance in the health system (Participant 5).*

Participants also perceive that the qualities beneficial for followership, such as assertiveness and communication, are better recognized and utilized in leadership roles. This perception influences their career aspirations, as they believe these traits are wasted in followership roles.

*I am a person who is much more assertive… I would say that I am capable of being a good leader (Participant 3).*

The desire to enact significant change further motivates individuals to pursue leadership, viewing it as the only effective position from which to influence organizational practices.

*To be a leader, definitely…You can make the change…As a staff, I will definitely not be able to make that change in the department. The leader is everything (Participant 4).*

Additionally, the transition into leadership roles is often associated with increased comfort and competence over time, suggesting a maturation path that favors leadership as careers progress.

*Well, at this point in my career, I'd say I'd rather play the leader… it's going to be hard to organize the work and deal with subordinates if you don't have enough experience (Participant 6).*

Even though leadership entails greater responsibilities, the perceived advantages and opportunities for impact make these positions more appealing.

*Despite the more responsibilities in the role of the leader… but I prefer this role (Participant 7).*

### 6.3 Lack of training and development opportunities

An analysis of participant feedback revealed systemic barriers that nurses face in accessing professional development, particularly programs that cultivate followership skills. This lack contributes significantly to the undervaluation of followership within the healthcare system.

*Actually, there is no such training that focuses on followership (Participant 2).*

*Training courses are mostly for leaders, but courses for subordinates are very few (Participant 5).*

*Priority is given to people in management positions such as heads of nursing who receive such courses (Participant 6).*

These insights collectively emphasize the organizational culture that disproportionately favors leadership development at the expense of comprehensive team growth.

### 6.4 Lack of collaboration

Participants identified poor collaboration, along with avoidance of responsibilities and punctuality issues, as major barriers to effective followership in healthcare settings. These challenges could compromise patient care and increase workloads for team members.

*Insufficient cooperation between colleagues may sometimes cause problems in patient care… Collaboration is essential to a team's success (Participant 7).*

### 6.5 Language barriers

The interviews revealed that language proficiency, particularly in English, poses a significant barrier to effective followership among healthcare professionals. This issue is especially acute for Saudi nurses with diploma qualifications, limiting their full engagement in their professional roles and impeding their contributions to team dynamics. Participants highlighted that inadequate English skills obstruct essential communication and collaboration within healthcare teams. The struggle with language proficiency not only affects the clarity of medical instructions but also hampers effective interaction among team members.

*I'm definitely struggling with English, though it's much better than it was at the beginning of my first year in the emergency department (Participant 4).*

*One of the Saudi nurses came to me to help him translate what the doctor had written in the patient's file (Participant 7).*

## 7 Facilitators of followership

Within this overarching theme, participants addressed some of the factors, skills, and characteristics or behaviors that they believed were important to facilitate effective followership in their workplaces. In the previous theme, participants spoke of barriers to followership that they felt were currently in place. When considering facilitators, the factors they considered were aspirational recognizing the need for change in their workplace.

### 7.1 Effective leadership

Effective leadership emerged as a crucial factor influencing followership within the workplace. Participants underscored that leadership qualities significantly impact team dynamics and effectiveness. Leadership effectiveness, as participants noted, directly affects the ability of followers to contribute meaningfully to team goals. Leaders who exhibit strong motivational qualities and set a positive example for their teams create environments that foster effective followership.

*I consider many factors, including the leader and whether he or she is a good or bad leader. It's about who's the leader in this area (Participant 4).*

The negative impact of inadequate leadership was also highlighted, as ineffective leaders often undermine team cohesion and productivity. Overall, the analysis points to a consensus among participants that the style and effectiveness of leadership are fundamental to creating an environment where effective followership can flourish.

*If the leader is not a good motivator for the team, and if the leader himself is not effective, then this will reflect negatively on the team and reduce the team's effectiveness level (Participant 5).*

### 7.2 Followership training

The significance of followership training emerged as a central theme, with participants highlighting the potential benefits of equipping followers with specialized skills. In healthcare organizations, where followers comprise the majority, training is perceived as crucial for fostering effective followership and enhancing overall team performance. Participants consistently expressed the view that specialized education empowers followers to contribute more meaningfully to team goals. They believe that such training would enable followers to complement leadership efforts and improve organizational outcomes.

*If there are special courses on followership, as we work as a team, the more knowledge the team has, the better the outcomes will be, because the leader is one or very few, while the followers are the majority. If they have [good] training and knowledge, they are the ones who can raise the performance of leaders and the organization as a whole (Participant 6).*

The need for formal training was also recognized within the healthcare context, where structured educational programs could help align the goals of staff and leaders.

*So, I think if the hospital could provide us with training for this, that will make it better for both the staff and the leaders (Participant 2).*

### 7.3 Experience

Clinical experience emerged as a pivotal factor in fostering effective followership within nursing teams. The consensus among participants is that experience bolsters confidence, enhances communication skills, and improves teamwork and collaboration.

*The first factor that makes you effective is the factor of experience…When you are involved in a system or in an emergency and lack experience, it will be difficult even to express an opinion…Experience makes you able to participate effectively with the team and the leader (Participant 1).*

*The experience, of course. It will have a huge effect on the quality of the practice (Participant 3).*

Furthermore, participants emphasized that clinical experience is more valuable than academic qualifications in this context. Practical exposure to real-world situations is perceived as instrumental in honing the skills required for effective followership.

*I think it's more about the experience, the clinical experience, not the qualifications (Participant 2).*

### 7.4 Communication skills

Communication skills were unanimously recognized as vital for effective followership in healthcare. Participants emphasized that clear and effective communication is crucial in fostering understanding and collaboration among nurses and with patients. Effective communication was seen as a cornerstone of teamwork and patient care, enabling nurses to work cohesively and align their efforts.

*I think the communication skills are very important (Participant 2).*

*The most important non-clinical skills for nurses is communication, which is an important skill to help nurses to understand each other and patients (Participant 6).*

Participants also detailed the multifaceted nature of communication, emphasizing the importance of both conveying information and active listening.

*Also, communication skills such as communicating in the department or communicating with team members effectively and listening to the instructions of the superiors with the possibility of exchanging views (Participant 7).*

### 7.5 Positive relationships, respect, and collaboration

Positive relationships, respect, and collaboration are essential components of effective followership in nursing. Participants emphasized that nurturing these elements directly influences job satisfaction, teamwork, and the quality of care delivery.

*Also, I think being friendly and having good relationships with your colleagues is very important to having a positive and friendly work environment (Participant 2).*

*The respect I gained from the institution is a very important and critical part of being satisfied with my job (Participant 3).*

*I mean the team members have to help each other, and the team work has to be complementary job (Participant 5).*

### 7.6 Understanding own and other's roles

Understanding one's role and the roles of others within a team is crucial for effective followership. Participants emphasized that role clarity is essential for patient care management and promoting teamwork. Participants noted that understanding the responsibilities of each team member enables effective collaboration and support.

*also, knowing your role and the roles of others, this thing is very important, and helps you to be effective with others greatly (Participant 5).*

*understanding the nature of the work of others in your team so that you can provide support and cooperation (Participant 6).*

Thus, when followers are aware of their roles and those of others, they can better anticipate needs, offer appropriate support, and contribute to a cohesive and efficient healthcare team.

### 7.7 Commitment

Commitment to work and patient care emerged as a crucial aspect of successful followership based on participants’ views. Participants emphasized that dedication to the health and safety of patients defines an effective follower in healthcare. Effective followers demonstrate strong commitment through punctuality, responsibility, and prioritizing patient care. This dedication was highlighted as integral to their role.

*Secondly, interest in work and commitment to the times of attendance and departure from work. Also caring for the patient. I have only been employed in this place for the sake of caring for the patient or for the patient's interest (Participant 7).*

*The best output is to give the patient the best care and management possible (Participant 3).*

### 7.8 Flexibility/Adaptability

Flexibility and adaptability were identified as key qualities of effective followership in nursing. Participants emphasized that these skills are essential for effectively navigating diverse situations and challenges, particularly in times of crisis.

*a good follower should always be resilient, self-motivated, self-reliant, independent, and, of course, flexible and adaptable to whatever changes or circumstances arise (Participant 3).*

## 8 Discussion

This qualitative study explored the nurses’ conceptualization of followership, their involvement in decision-making as followers, and the barriers and facilitators of effective followership from their perspectives.

The participants' limited acquaintance with followership was anticipated and corresponds with the prevalent literature that characterizes followership as an overlooked and insufficiently researched topic (Spriggs, 2016; Everett, 2016; Leung et al., 2018; Carsten et al., 2010; Uhl-Bien et al., 2014; Loyola and Aiswarya, 2023). The participants' perception of followership primarily revolved around competencies related to teamwork and the interplay between followers and leaders for the coordination of duties. The essential elements of followership, such as courage, assertiveness, the ability to speak up, voice opinions, and offer constructive criticism to leaders as needed, the capacity to reduce hierarchical structures, and the adoption of moral positions, were not initially included in their conceptualization of followership. These elements, among others, have been recognized in prior research as significant aspects of followership (Kelley, 1992; Chaleff, 2009; Carsten et al., 2010; Hay-David et al., 2022; Weber, Bunin, and Hartzell, 2022). For instance, when the participants were asked about what makes a good follower, or facilitator and what are the barriers to effective followership they identified factors that were more associated with teamwork rather than followership such as time management, cooperation and positive relationships, understanding of team roles, work-life balance challenges, language and understaffing barriers. Although such factors are important in increasing or decreasing work productivity and satisfaction, they are not necessarily related to followership. For example, time management, work-life balance and understaffing would not be factors that are relevant or directly affect exemplary followership or the individual's style of being a good follower. In the literature we find, that “a good follower is more like a partner, sharing a common vision with the leader, working actively to achieve it while also raising any concerns. Good followership requires judgment, competence, work ethics, honesty, courage, loyalty, discretion, and ego management” (Pathak and Wong, 2022, p.740). Although the participants identified other factors that related to followership such as leadership as an important barrier to or facilitator of exemplary followership, decision-making, effective communication, flexibility and commitment, but mostly they overlapped the concepts of teamwork or other work-related factors with followership. Hence, it is important to incorporate followership concepts into nursing education and continuous development training programs to increase nurses’ knowledge and understanding of followership and its impact on clinical practice and patient safety.

The participants' involvement in decision-making was highly influenced by hierarchy and the power disparities that existed between nurses and their superiors or between nurses and physicians. This was consistent with earlier research that classified Saudi Arabia's organizational culture as one of a high-power distance (Hofstede, Hofstede, and Minkov, 2010), in which hierarchy dominates and followers have limited participation in decision-making (Schuder, 2016). The hierarchical relationships and power imbalances between leaders and followers were reflected in some participants' language. This was evidenced by using terms such as 'boss' to refer to leaders and 'subordinate' to describe staff members. The participants’ accounts revealed that followers' ineffective or limited involvement in decisions over patient care has resulted in some clinical errors and a sense of job dissatisfaction. The narratives provided by the participants pertaining to the impact of leadership on their effectiveness and on patient safety and their frustration about some poor leadership practices showed that leadership was a major barrier to exemplary followership. These narratives indicated that autocratic leadership practices, as opposed to transformational ones, are commonly used at their workplace. According to Robbins and colleagues, “An autocratic style is that of a leader who typically tends to centralize authority, dictate work methods, make unilateral decisions and limit employee participation” (2021, p.329). This further signifies that the enactment of exemplary followership would be greatly influenced by the specific style of leadership present within the organizational context (Robbins et al., 2021). Thus, enhancing and encouraging the knowledge and practice of transformational leadership and exemplary followership could be potential solutions for preventing unsafe practices and enhancing followers’ satisfaction levels in the workplace (Pathak and Wong, 2022; Robbins et al., 2021; Hay-David et al., 2022; Adams and Gibson, 2024).

Another major barrier to followership was that the followership role or being a follower was perceived as unvalued compared to being a leader. The participants' responses pertaining to their preferences for assuming the leader and follower roles substantiated the notable power disparities inherent in these roles. This is because people "are socialized to view hierarchical systems such as organizations in terms of the status inequalities and power differentials that exist between individuals in various hierarchical positions” (Carsten et al., 2010, p.546). Five participants expressed a preference for assuming the leadership role to gain more power, influence, and access to training and career development opportunities, as these incentives were very limited in the followership role. For some participants, assuming the role of a follower was associated with reduced responsibility, as the burden of responsibility was commonly attributed to the role of the leader rather than the follower. This notion indirectly implies that the role of followers is perceived as unimportant or of lesser value than the role of leaders, potentially impeding the development of exemplary followership practices. This also accords with Carsten et al., (2010), that "the image that followers are less responsible, accountable, and effectual than leaders is reinforced by a top-down approach to leadership that is grounded in hierarchical notions that status, power, influence, and prestige are reserved for those at the upper echelon" (2010, p.546). Some participants reported having exemplary followership qualities such as assertiveness and communication skills but seemed convinced that such qualities are more associated with the leader role than the follower role. The participants didn't appear to comprehend that the qualities required for effective followership are actually the same as those required for effective leadership (Kelley, 1988 and 1992; Chaleff, 2009; Everett, 2016; Carsten et al., 2010). The nurses involved in this study showed typical perspectives regarding the concepts of leadership and followership. Consistent with prior research, healthcare professionals, including nurses, doctors, and pharmacy clinicians, conceptualized followership in a negative light when compared with leadership (Barrow, McKimm, and Gasquoine, 2011; Gordon et al., 2015; Dikun et al., 2021). Therefore, it is important to increase awareness among healthcare professionals, both followers and leaders, about the importance of the follower role in healthcare. In addition, enhancing the equality between the leader and follower roles in terms of training, development opportunities, and incentives and using the concepts of exemplary followership and transformational leadership that allows and encourages exemplary followership practices could aid in the redefinition of the two roles, thereby achieving a state of balance that is characterized by complementarity and partnership rather than hierarchy. In this regard, the recommendations made to facilitate effective followership in the surgical field can be of benefit as they are also applicable to the nursing profession. They include “1) redefine and reinforce followership and leadership roles as equal but different activities; 2) teach essential skills such as critical thinking, discretion, adaptability, courage, loyalty, and ego management and well-balanced inter-personal communication to promote exemplary followership; 3) evaluate surgeons’ performance on the basis of their followership capacities; and 4) build organizational structure that encourages followership” (Pathak and Wong, 2022, p.741).

### 8.1 Limitations

The present study acknowledges certain limitations that merit consideration. Firstly, the recruitment of seven nurses from four health regions in Saudi Arabia, while deemed sufficient for achieving data saturation, presents a challenge in terms of representing the diverse experiences and perceptions across the broader nursing population in Saudi Arabia. Additionally, the gender and national representation within the participant pool were limited, with a predominance of male nurses and only one expatriate nurse participating. This composition might have influenced the thematic insights, particularly regarding perceptions of followership, leadership, and professional dynamics in the context of Saudi Arabian healthcare settings. Future studies could aim for a more balanced gender and cultural representation to explore these dynamics further. Despite these limitations, this study offers valuable insights into the concept of followership within the context of nursing in Saudi Arabia, a topic that has been scarcely explored in the existing literature. This study should not be viewed as an isolated examination of followership in Saudi Arabia but rather as an integral component of a mixed-methods research project.

## 9 Conclusion and implications

Our study delves into the intricate landscape of followership within the nursing profession in Saudi Arabia, uncovering themes that illuminate both the challenges and opportunities within this domain. The nuanced understanding of followership as distinct from, yet integral to, effective teamwork addresses a critical gap in existing research, providing a fresh lens through which to view the dynamics of healthcare teams. The identification of hierarchical and autocratic leadership as barriers to nurse involvement in decision-making highlights a pivotal area for managerial intervention. It also suggests the need for transformative leadership practices that empower nurses, especially within high-power distance cultures like Saudi Arabia. Furthermore, our exploration of the barriers to and facilitators of effective followership opens new pathways for enhancing nursing education and professional development programs, emphasizing the need to integrate followership concepts into nursing curricula and continuous professional development. Early exposure to and training in followership competencies can equip nurses with a balanced understanding of their roles as followers and leaders, enhancing their contribution to patient care and team dynamics.

The themes we have uncovered, while rooted in the Saudi Arabian context, speak to universal aspects of the healthcare experience, transcending cultural and geographical boundaries. The undervaluation of the follower role, for instance, reflects a widespread misunderstanding (Barrow, McKimm, and Gasquoine, 2011; Gordon et al., 2015; Dikun et al., 2021) that may hinder the efficacy of healthcare teams worldwide. By addressing this misconception, our study advocates for a global healthcare dialogue that repositions followership as a critical component of team success, paving the way for more cohesive and effective healthcare delivery systems across cultures. Additionally, the constraints on nurse participation in decision-making processes due to rigid leadership structures are challenges faced by healthcare systems around the world. Our findings offer a call to action for healthcare leaders globally to embrace more inclusive leadership models that empower nurses and other healthcare professionals. This approach acknowledges the significant role of followers in healthcare teams, which can lead to better patient outcomes and workplace satisfaction. Ultimately, this study contributes to the sparse literature on followership in nursing, particularly within a high-power distance culture, and lays the groundwork for further research to explore the nuances of followership across different cultural and organizational contexts. This will assist in developing universally applicable strategies for fostering effective followership in healthcare.

## Ethics statement

Informed consent for participation was obtained from all the participants. This study was approved by the University of Adelaide Human Research Ethics Committee (approval number: H‐2020‐026) and the Central Institutional Review Board at the Ministry of Health in Saudi Arabia (log number: 20‐161E).

## Ethical approval

This study was approved by The University of Adelaide Human Research Ethics Committee (approval number: H-2020-026) and the Central Institutional Review Board at the Ministry of Health in Saudi Arabia (log number: 20-161E).

## Authorship statement

We confirm that all listed authors meet the authorship criteria and that all authors are in agreement with the content of the manuscript.

## CRediT authorship contribution statement

**Sulaiman Alanazi:** Writing – original draft, Visualization, Validation, Methodology, Investigation, Formal analysis, Data curation, Conceptualization. **Richard Wiechula:** Writing – review & editing, Validation, Supervision, Methodology, Conceptualization. **David Foley:** Writing – review & editing, Validation, Supervision, Methodology, Conceptualization.

## Declaration of competing interest

The authors declare no conflict of interest.

## References

[bib0001] Abdel-Malak R. (2016). A concept analysis of “Follower” within the context of professional nursing. Nurs. Forum..

[bib0002] Adams T., Gibson A. (2024). Followership: an undervalued concept in effective teams within the military and NHS. BMJ Mil. Health.

[bib0003] Alanazi S., Wiechula R., Foley D. (2022). Followership in nurses working in Saudi Arabian hospitals: a cross-sectional study. Nurs. Forum..

[bib0004] Alanazi S., Wiechula R., Foley D. (2023). Followership in health care clinicians: a scoping review. JBI. Evid. Synth..

[bib0005] Almalki M., FitzGerald G., Clark M. (2011). The nursing profession in Saudi Arabia: an overview. Int. Nurs. Rev..

[bib0006] Andersen P.O., Jensen M.K., Lippert A., Østergaard D. (2010). Identifying non-technical skills and barriers for improvement of teamwork in cardiac arrest teams. Resuscitation.

[bib0007] Barrow M., McKimm J., Gasquoine S. (2011). The policy and the practice: early-career doctors and nurses as leaders and followers in the delivery of health care. Adv. Health Sci. Educ. Theory. Pract..

[bib0008] Bould M.D., Sutherland S., Sydor D.T., Naik V., Friedman Z. (2015). Residents’ reluctance to challenge negative hierarchy in the operating room: a qualitative study. Can. J. Anaesth..

[bib0009] Braun V., Clarke V. (2006). Using thematic analysis in psychology. Qual. Res. Psychol..

[bib0010] Bryman A. (2004). Qualitative research on leadership: a critical but appreciative review. Leadersh. Q..

[bib0011] Can A., Aktaş M. (2012). Cultural values and followership style preferences. Procedia Soc. Behav. Sci..

[bib0012] Carsten M., Uhl-Bien M., West B., Patera J., McGregor R. (2010). Exploring social constructions of followership: a qualitative study. Leadersh. Q..

[bib0013] Chaleff I. (2009).

[bib0014] Chaleff I. (2016). In praise of followership style assessments. J. Leadersh. Organ. Stud..

[bib0015] Conger J. (1998). Qualitative research as the cornerstone methodology for understanding leadership. Leadersh. Q..

[bib0016] Crawford J., Daniels M. (2014). Follow the leader: how does “followership” influence nurse burnout?. Nurs. Manage.

[bib0017] Crossman B., Crossman J. (2011). Conceptualising followership: a review of the literature. Leadership.

[bib0018] Dikun J., Bouldin A., Holmes E., Rosenthal M. (2022). A qualitative approach to investigating developmental opportunities among leaders and followers. Am. J. Pharm. Educ..

[bib0019] Everett L. (2016). Academic-practice partnerships: the interdependence between leadership and followership. Nurs. Sci. Q..

[bib0020] Fadden S., Mercer S. (2019). Followership in complex trauma. Trauma.

[bib0021] Freeman M. (2021). Dispelling the myths of followership in nursing. Can. J. Nurs. Res..

[bib0022] Gordon L., Rees C., Ker J., Cleland J. (2015). Dimensions, discourses and differences: trainees conceptualising health care leadership and followership. Med. Educ..

[bib0023] Green B., Oeppen R., Smith D., Brennan P. (2017). Challenging hierarchy in healthcare teams – ways to flatten gradients to improve teamwork and patient care. Br. J. Oral Maxillofac. Surg..

[bib0024] Hay-David A., Herron J., Gilling P., Brennan P. (2022). Assertive followership: how to make a team safer. Br. J. Oral Maxillofac. Surg..

[bib0025] Hinshaw K. (2016). Human factors in obstetrics and gynaecology. Obstet. Gynaecol. Reprod. Med..

[bib0026] Hofstede G., Hofstede J., Minkov M. (2010).

[bib0027] Honan D., Lasiuk G., Rohatinsky N. (2022). A scoping review of followership in nursing. Nurs. Leadersh..

[bib0028] Kellerman B. (2008).

[bib0029] Kelley R. (1988). In praise of followers. Harv. Bus. Rev..

[bib0030] Kelley R. (1992).

[bib0031] King N. (2004). Essential Guide to Qualitative Methods in Organizational Research.

[bib0032] Leung C. (2018). Followership: a review of the literature in healthcare and beyond. J. Crit. Care.

[bib0033] Lincoln Y., Guba E. (1985).

[bib0034] Lopez S., Freeman M. (2018). Refocusing nursing's lens on followership. Nurs. Leadersh..

[bib0035] Loyola M., Aiswarya B. (2023). Followership in organisational leadership studies: a systematic literature review. Col. Bus. J..

[bib0036] Martin R. (2015). A review of the literature of the followership since 2008: the importance of relationships and emotional intelligence. Sage Open.

[bib0037] Ministry of Health, Saudi Arabia., 2020. *Annual statistical book*. Retrieved from https://www.moh.gov.sa/en/Ministry/Statistics/book/Documents/ANNUAL-STATISTICAL-BOOK-1438H.pdf [Accessed 27 February 2023].

[bib0038] Nowell L., Norris J., White D., Moules N. (2017). Thematic analysis: striving to meet the trustworthiness criteria. Int. J. Qual. Methods.

[bib0039] Pathak A., Wong A. (2022). Followership: the missing link in surgical leadership. Ann. Surg..

[bib0040] Riggio, R., 2020. Why followership? New Dir. Stud. Leadersh.. 167, 15–22. 10.1002/yd.20395. 32830922

[bib0041] Robbins S., Coulter M., DeCenzo D., Woods M. (2021).

[bib0042] Schuder K. (2016). Using followership to develop new leadership in cultures with greater power distance. J. Leadersh. Organ. Stud..

[bib0043] Sculli G. (2015). Effective followership: a standardized algorithm to resolve clinical conflicts and improve teamwork. J. Healthc. Risk. Manage.

[bib0044] Spriggs D. (2016). Followership: a critical shortfall in health leadership. Intern. Med. J..

[bib0045] Uhl-Bien M., Riggio R., Lowe K., Carsten M. (2014). Followership theory: a review and research agenda. Leadersh. Q..

[bib0046] Weber L., Bunin J., Hartzell J. (2022). Building individual and organizational wellness through effective followership. J. Healthc. Leadersh..

[bib0047] Whitlock J. (2013). The value of active followership. Nurs. Manage.

